# Modelling Skeletal Muscle Ageing and Repair *In Vitro*

**DOI:** 10.1155/2023/9802235

**Published:** 2023-07-04

**Authors:** Janelle Tarum, Hans Degens, Mark D. Turner, Claire Stewart, Craig Sale, Lívia Santos

**Affiliations:** ^1^Department of Sport Science, Sport Health and Performance Enhancement Research Centre (SHAPE), School of Science and Technology, Nottingham Trent University, Nottingham, UK; ^2^Musculoskeletal Science and Sports Medicine Research Centre, Department of Life Sciences, Manchester Metropolitan University Institute of Sport, Manchester Metropolitan University, Manchester, UK; ^3^Lithuanian Sports University, Kaunas, Lithuania; ^4^Centre for Diabetes, Chronic Diseases and Ageing, School of Science and Technology, Nottingham Trent University, Clifton, UK; ^5^Research Institute of Sport and Exercise Science, Liverpool John Moores University, Liverpool, UK; ^6^Institute of Sport, Manchester Metropolitan University, Manchester, UK

## Abstract

Healthy skeletal muscle can regenerate after ischaemic, mechanical, or toxin-induced injury, but ageing impairs that regeneration potential. This has been largely attributed to dysfunctional satellite cells and reduced myogenic capacity. Understanding which signalling pathways are associated with reduced myogenesis and impaired muscle regeneration can provide valuable information about the mechanisms driving muscle ageing and prompt the development of new therapies. To investigate this, we developed a high-throughput *in vitro* model to assess muscle regeneration in chemically injured C2C12 and human myotube-derived young and aged myoblast cultures. We observed a reduced regeneration capacity of aged cells, as indicated by an attenuated recovery towards preinjury myotube size and myogenic fusion index at the end of the regeneration period, in comparison with younger muscle cells that were fully recovered. RNA-sequencing data showed significant enrichment of KEGG signalling pathways, PI3K-Akt, and downregulation of GO processes associated with muscle development, differentiation, and contraction in aged but not in young muscle cells. Data presented here suggest that repair in response to *in vitro* injury is impaired in aged vs. young muscle cells. Our study establishes a framework that enables further understanding of the factors underlying impaired muscle regeneration in older age.

## 1. Introduction

Skeletal muscle regeneration is a complex and finely regulated biological process that shares molecular and cellular aspects with embryonic development [1–3]. This homeostatic process is made possible by the coordinated cooperation between different cell populations, such as satellite cells (mitotically quiescent stem cell population residing between the sarcolemma and basal lamina), fibroblasts, inflammatory cells, and the myofiber microenvironment, all decisive to the adequate regeneration of the muscle tissue [4–7]. The role of satellite cells in muscle regeneration has been extensively studied. Upon injury, they exit their quiescent and initiate proliferation. This is mediated by a rapid activation of the Notch signalling pathway, upregulation of cyclin D1, and suppression of the TGF-*β*-Smad3 signalling pathway [8]. After generating enough progeny, the cells exit the proliferative state to initiate differentiation where they fuse into the injury site [9].

Although muscle exhibits a robust regeneration capacity, this becomes impaired with ageing contributing to the decline of musculoskeletal health, which, in turn, is a contributory factor in the development of sarcopenia [10]. This impairment is partially due to the limited proliferative capacity of satellite cells on account of Notch and Cyclin D1 downregulation and a suboptimal myofiber niche created by chronic inflammation—a hallmark of the ageing muscle [11–13].

It is vital to understand the muscle regenerative response in the context of ageing since it has been suggested that an accumulation of contraction-induced microdamage that is not adequately repaired may be one of the causes of muscle wasting, sarcopenia, and anabolic resistance typically observed in older people [14]. Yet, there exists no *in vitro* human model to enable such investigations. Therefore, the aim of this study was to develop an *in vitro* human muscle regeneration model and characterise the changes in morphology, cell cycle, and mRNA transcription during recovery from damage in young and aged muscle.

To generate this model, we derived myotubes (differentiated muscle cells) from myoblasts isolated from young and older individuals and provoked an injury using the muscle toxin barium chloride (BaCl_2_) that is commonly used to induce injury to examine muscle repair and regeneration [15]. BaCl_2_ destroys the cytoskeleton without abrogating the cell's regeneration capacity [16–18] and thus can be used to enhance our understanding of myotube regeneration and underlying nuclear messaging occurring during this process.

The proposed muscle regeneration model could be easily transformed into a reproducible high-throughput application. If successful, this high-throughput model will have significant application in studies investigating muscle regeneration and ageing, sarcopenia, metabolic and genetic diseases, as well as for initial drug discoveries.

## 2. Methods

### 2.1. Experimental Design

**Figure 1:**
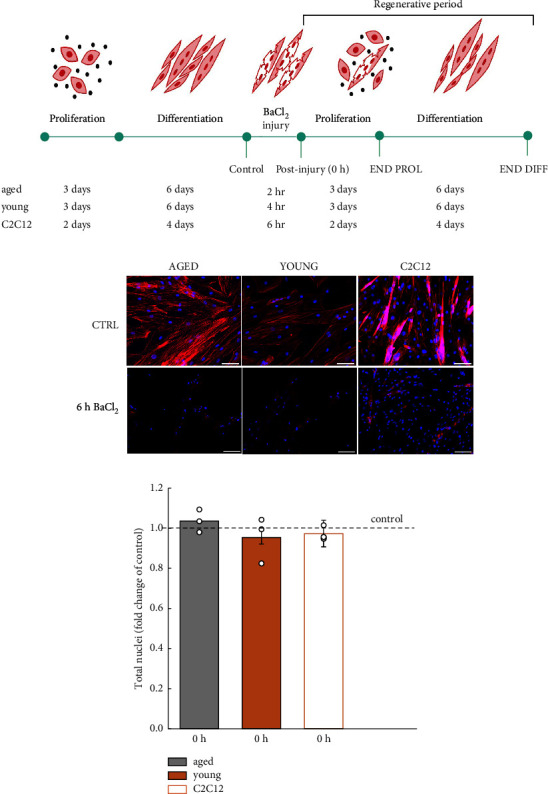
Human muscle cells are more sensitive to barium chloride-induced injury than mouse muscle cells. Experimental design: myotubes derived from mouse (C2C12) and young and aged human myoblasts were chemically injured with 12% BaCl for 6, 4 and 2 h, respectively and then allowed to regenerate (a). Immunofluorescence images showing F-actin and nuclei labelling with phalloidin and DAPI of C2C12, young, and aged human donors before injury (CTRL) and when incubated for 6 h with BaCl_2_. Scale bars represent 100 *μ*m (b). No significant difference in the total nuclei number was found before (CTRL) and after injuring the mouse (C2C12) and young and aged human myotubes with 12% BaCl_2_ for 6, 4 or 2 h, respectively; Circles indicate individual data points of technical replicates (c).

The experimental design is illustrated in Figure 1(a). The acute effects of BaCl_2_ exposure on skeletal muscle regeneration were studied using a modified experimental design previously described by Fleming et al. [19]. After human or mouse myoblasts became confluent and differentiated into myotubes, they were injured by exposure to the toxin BaCl_2_. After injury, cells underwent a proliferation and differentiation period that replicated the preinjury protocol. Three-four independent experiments were obtained from each cell line.

### 2.2. Mouse Myoblast Culture

C2C12 murine myoblasts (LGC standards/ATCC) were cultured in 6-well plates at a density of 3000 cells/cm^2^ and incubated in a humidified 5% CO_2_ atmosphere at 37°C in the growth medium (GM) composed of high-glucose Dulbecco's Modified Eagle Medium (DMEM, Sigma) supplemented with 1% penicillin-streptomycin solution (Invitrogen) and 10% fetal bovine serum (FBS, Gibco). Myoblasts were cultured to ∼90% confluence before GM was replaced with the low-serum differentiation medium (DM), composed of high-glucose DMEM plus 2% horse serum and 1% penicillin-streptomycin solution. The total culture period was 2 days in GM followed by a further 4 days in DM for myotube formation. All experiments were performed at passages below 10.

### 2.3. Human Myoblast Culture

Human skeletal muscle cells (aged donor (68 yrs, male) purchased from Promocell and young donor (20 yrs, male), purchased from Lonza were seeded at 3500 cells/cm^2^ onto 35 mm 6-well plates and grown in GM (low-glucose DMEM with 20% FBS and 1% penicillin-streptomycin solution). At 90% confluence, GM was replaced with DMEM containing 2% horse serum and 10 *μ*g/mL insulin (Sigma) to induce myogenic differentiation. The total culture period was 3 days in GM followed by a further 6 days in DM. All experiments were performed at passages 3–5.

### 2.4. Cell Injury

Myotubes were differentiated from human and mouse myoblasts as detailed above until reaching confluency. Fresh DM was added to all cell lines before inducing injury. Then, 50 *μ*L/mL of 12% w/w BaCl_2_ solution was added to the medium, followed by a 2 h, 4 h, or 6 h incubation for C2C12, young, and aged human myotubes, respectively, to cause injury. Myotubes derived from the C2C12 cell line were exposed to BaCl_2_ for 6 h, as detailed in the literature [19]. Initially, we also exposed human myotubes for 6 h to BaCl_2_, but this caused complete removal of the nuclei, in addition to the cytoskeleton injury, and resulted in an inability to repair the injury (Figure 1(b)). This prompted us to identify the optimal injury time, defined as the period of BaCl_2_ exposure that caused damage to the cytoskeleton while maintaining the number of nuclei constant (Figure 1(c)), for each cell line. To identify this optimal period, we stained the cytoskeleton with phalloidin and the nuclei with DAPI and quantified the nuclei number (Figures 1(b) and 1(c)). We showed that the optimal exposure duration was 6 h for C2C12 and 4 h and 2 h for young and aged human-derived myotubes. After incubation with BaCl_2_, cultures were washed with phosphate-buffered saline (PBS) (Sigma) to remove residual BaCl_2_ containing media. Preinjury (CTRL) and directly postinjury (0 h) samples were collected at the end of BaCl_2_ incubation. Injury was followed by a regenerative period that follows the exact protocol used to differentiate cultures. During regeneration, cells for morphological and transcriptome analysis were collected: on day 2 (END PROL) and day 7 (END DIFF) for C2C12 and on day 3 (END PROL) and day 9 (END DIFF) for human cells.

### 2.5. Cell Proliferation

To assess cell proliferation, the Click-IT®Plus EdU Imaging Kit (Invitrogen) was used. Stock solutions were prepared as indicated by the manufacturer. 2x working solution of 10 mM EdU was added to prewarmed culture medium (GM or DM, depending on the timepoint) and incubated for 4 h under optimal growth conditions (humidified incubator 37°C, 5% CO_2_). After incubation, cells were washed 2x with PBS followed by immunostaining protocol.

### 2.6. Immunostaining

Cells were fixed using 10% formaldehyde and permeabilised with 0.25% Triton X-100 (Sigma-Aldrich) solution. The actin cytoskeleton of cells was identified using Alexa Fluor 568 phalloidin conjugate (1 : 40, Invitrogen), and Prolong Gold Antifade Mountant with DAPI (Invitrogen) was used to counterstain myonuclei. To further assess the cell dynamics postinjury, EdU assay (Click-iT Plus EdU Imaging kit, Invitrogen) was applied to cultures marking the proliferative cells. In brief, 30-minute incubation with Click-It Plus reaction cocktail consisting of 1x reaction buffer, copper protectant, reaction buffer additive, and Alexa Fluor Picolyl Azide was performed at room temperature in the dark. Cells were then washed with PBS. Myonuclei were labeled with Hoechst staining for 30 min.

All fluorescence staining was performed before injury (CTRL), postinjury (0 h), at the end of proliferation (END PROL), and at the end of differentiation (END DIFF) timepoints during the regenerative period.

### 2.7. Image Acquisition, Processing, and Analysis

Cells were visualised under a fluorescence microscope (Olympus), and image acquisition performed using Leica Microscope software. At least 10 fields within each well were captured and approximately 180 myotubes from each condition were included in the analysis. Myogenic fusion index was calculated as the ratio of the nuclei number in myotubes with two or more nuclei versus the total number of nuclei. To quantify the percentage of cells synthesising DNA, the ratio between EdU + cells and total myonuclei was calculated. General image analysis was performed using ImageJ software v2.3.0 (NIH, U.S).

### 2.8. RNA Extraction

Myoblasts/myotubes were collected at three timepoints across the experiment: at control (CTRL), at the end of proliferation (END PROL), at the and end of differentiation (END DIFF) during the regenerative period and transferred to low binding/RNase free microcentrifuge tubes containing 350 *μ*L RNeasy lysis buffer (Qiagen) supplemented with *ß*-mercaptoethanol (Sigma-Aldrich) to isolate total RNA using RNeasy mini kit (Qiagen). Following lysis, samples were homogenised and loaded onto RNeasy silica membrane for RNA binding. Concentrated and pure RNAs were eluted in RNase-free water. Concentrations were quantified using spectrophotometry NanoDrop 2000, Thermo Fisher Scientifc refer to the details of an equipment to assess RNA quality. It is not a citation and should be mantained as it is.

### 2.9. RNA Sequencing

RNA integrity was assessed using the RNA Nano 6000 Assay Kit of the Bioanalyzer 2100 system (Agilent Technologies, CA, USA). Messenger RNA was purified from total RNA using poly-T oligo-attached magnetic beads. To select cDNA fragments of preferentially 370∼420 bp in length, the library fragments were purified with the AMPure XP system (Beckman Coulter, Beverly, USA). Then, PCR was performed with Phusion High-Fidelity DNA polymerase, Universal PCR primers, and Index (X) Primer. At last, PCR products were purified (AMPure XP system) and library quality was assessed on the Agilent Bioanalyzer 2100 system. After cluster generation (cBot Cluster Generation System), the library preparations were sequenced on an Illumina Novaseq platform and 150 bp paired-end reads were generated. Differential expression analysis of two groups was performed using the DESeq2 R package (1.20.0). The resulting *p* values were adjusted using Benjamini and Hochberg's approach for controlling the false discovery rate. Genes with an adjusted *P* < 0.05 found by DESeq2 were assigned as differentially expressed. Four independent experiments were performed.

### 2.10. Pathway Analysis

GO enrichment analysis was calculated using GOseq R package, and KOBAS software was implemented for enrichment analysis in KEGG pathways. Terms with adjusted *p* values less than 0.05 were considered significantly enriched by differentially expressed genes. GO and KEGG pathway images were generated using the same software.

### 2.11. Statistical Analysis

The data were generally presented as mean ± the standard error of the mean (SEM). Statistical analysis was conducted using Graph Pad Prism for Mac Version 9.04. One-way ANOVA with Dunnet post hoc tests was used to analyse the total myonuclei number and EdU + cell data. Fusion index and myotube diameter were compared to preinjury values by using two-sided unpaired *t*-tests. *p* values <0.05 were considered statistically significant and were indicated within figures as ^*∗*^*p* < 0.05, ^*∗∗*^*p* < 0.01, and ^*∗∗∗*^*p* < 0.001.

## 3. Results

### 3.1. Human Muscle Cells Are More Sensitive to Barium Chloride-Induced Injury than Mouse Muscle Cells

Previous studies reported the development of injury models using 12% BaCl_2_ for 6 h in myotubes derived from the C2C12 mouse cell line. Since it is well established [18–20], we decided to utilise the C2C12 cell line in our experiments for comparative purposes. We were able to confirm that 12% BaCl_2_ for 6 h adequately removed the cytoskeleton without affecting the number of nuclei in the myotubes. However, when we incubated human myotubes under the same conditions, we discovered that such exposure time was causing excessive destruction of the cell structure and cell death (Figure 1(b)). Reducing the exposure time, we identified the optimal incubation period −2 h for myotubes derived from old and 4 h for cells derived from young myoblasts. These exposure times were deemed optimal as they caused cytoskeletal removal without significantly changing the total myonuclei number (Figure 1(c)).

### 3.2. Young and Aged Muscle Cells Show a Similar Number of EdU + Cells during the Proliferation Phase

**Figure 2:**
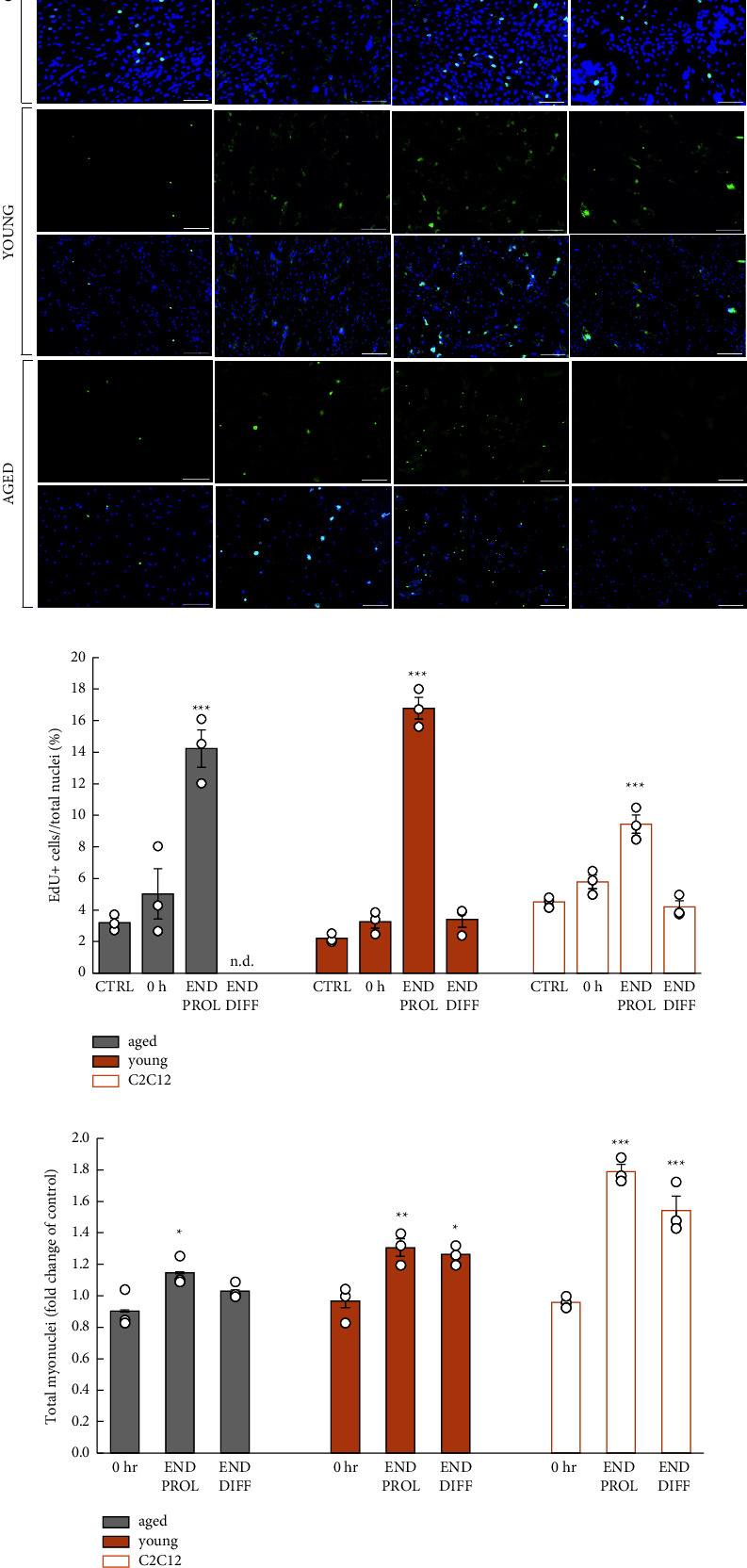
Young and aged muscle cells show similar proliferation response. EdU incorporation in mouse C2C12 and human skeletal muscle cells (EdU + cells are represented in green (arrow) and Hoechst + cells are represented in blue) indicating DNA synthesis in S-phase of cell cycle before (CTRL) and after BaCl_2_-induced injury. No EdU + cells were detected in old myotubes at the end of the regeneration period (END DIFF, n.d.) (a). Significant increase in EdU + cells in culture was reached by the end of proliferation of the regeneration period (END PROL) in each cell line (*n* = 3; ^*∗∗∗*^*p* < 0.001) (b). Total nuclei number in myotubes derived from mouse (C2C12) and human young and aged myoblasts after injury (0 h) and the regenerative period (END PROL and END DIFF). Treatment with BaCl_2_ retains precursor cells and initiates regenerative response in cultures. Significant increase in the total nuclei number was reached by the end of proliferation of the regeneration period (END PROL) in each cell line (*n* = 3; ^*∗*^*p* < 0.05, ^*∗∗*^*p* < 0.01, and ^*∗∗∗*^*p* < 0.001 (c). Statistical analysis performed by one-way ANOVA; all error bars represent standard error of the mean (SEM); scale bars 100 *μ*m; circles indicate individual data points of technical replicates.

The proliferation activity of muscle cells was assessed by EdU (5-ethynyl-2′-deoxyuridine) incorporation, which detects the cells entering the S-phase (Figure 2(a)). The number of EdU + cells increased significantly during the proliferation phase in muscle cells derived from both young and aged donors, referred to henceforth as young and aged muscle cells (Figure 2(b)). By the end of the regenerative phase, the number of EdU + cells declined in young and aged cells.

Total myonuclei number increased in human cells during proliferation and remained significantly elevated in young cells by the end of the differentiation period in relation to preinjury (Figure 2(c)).

Mouse myotubes derived from C2C12 showed significant increase in EdU + cells during the proliferation phase (Figure 2(b)) from baseline and a significant increase in myonuclei number during proliferation and after the differentiation phase (Figure 2(c)).

### 3.3. Aged Human Cells Show Impaired Differentiation during Regeneration

**Figure 3:**
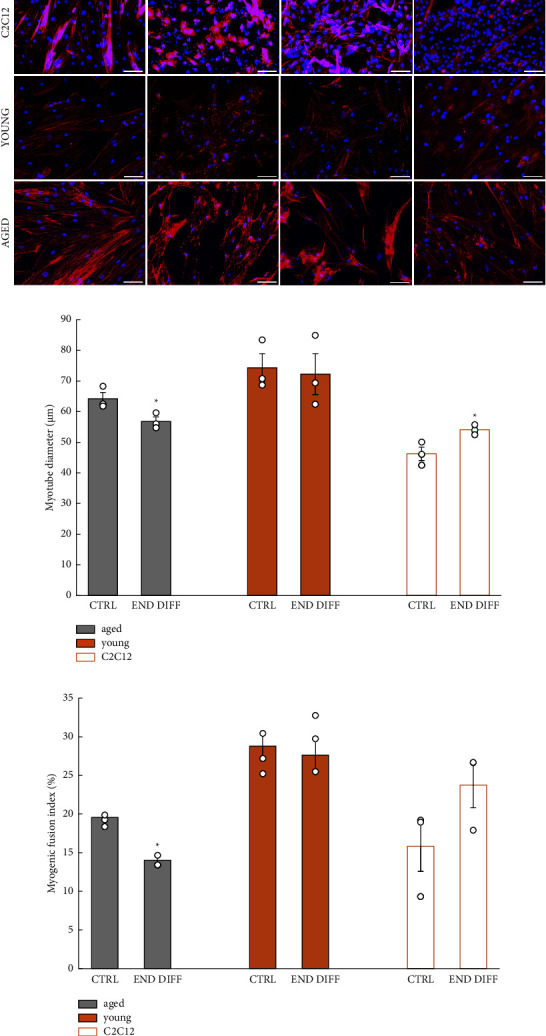
Aged muscle cells show reduced myotube diameter and fusion index after chemical insult with BaCl_2_. Immunofluorescence images showing recovery timepoints after BaCl_2_-induced injury in mouse C2C12 and young- and old-derived human myoblasts. Immunostaining for F-actin (red, phalloidin) and nuclei (blue, DAPI) show removal of myotubes but preserved precursor cells in response to chemical injury (a). Myotube diameter recovered to preinjury (CTRL) size in young and mouse C2C12, but remained significantly smaller in myotubes from older donors (*n* = 3, unpaired *t*-test. ^*∗*^*p* < 0.05) (b). Myogenic fusion index was significantly smaller in aged myotubes, but not in mouse C2C12 and young cells at the end of the regeneration period from the baseline (*n* = 3; unpaired *t*-test;^*∗*^*p* < 0.05) (c) (all error bars represent standard error of the mean (SEM); scale bars 100 *μ*m; circles indicate individual data points of technical replicates).

Then, we assessed myotube width and fusion which are important functional indicators of muscle cell differentiation (Figure 3(a)). We showed that young muscle cells recovered myotube width (Figure 3(b)) and fusion index (Figure 3(c)) to preinjury values. In contrast, aged cells did not exhibit the same levels of recovery, as evidenced by smaller myotube diameter (Figure 3(b)) and fusion index (Figure 3(c)). Mouse muscle cells showed a slight increase in myotube width and fusion index by the end of the self-repair process compared to preinjury (Figures 3(b) and 3(c)).

### 3.4. Cell Cycle and PI3k-Akt Signalling Pathways Are Significantly Enriched in Both Young and Aged Muscle Cells during the Proliferative Stage of Regeneration

**Figure 4:**
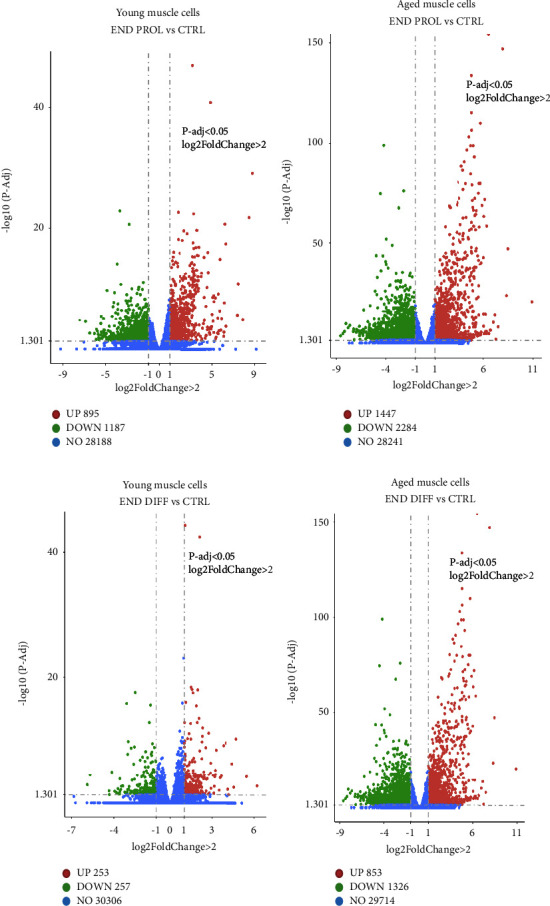
Volcano plots capturing significantly upregulated and downregulated genes in human muscle cells during regeneration vs. baseline. Volcano plots of young (a) and aged (b) muscle cells showing the number of upregulated and downregulated gene transcripts at the end of proliferation in the regeneration period (END PROL) compared to baseline and at the end of the regenerative process in young (c) and aged (d) muscle cells vs. control. Red and green dots indicate significantly upregulated and downregulated genes between timepoints, respectively. Blue dots denote genes not differentially expressed genes in END PROL or END DIFF vs. control. *n* = 4.

**Figure 5:**
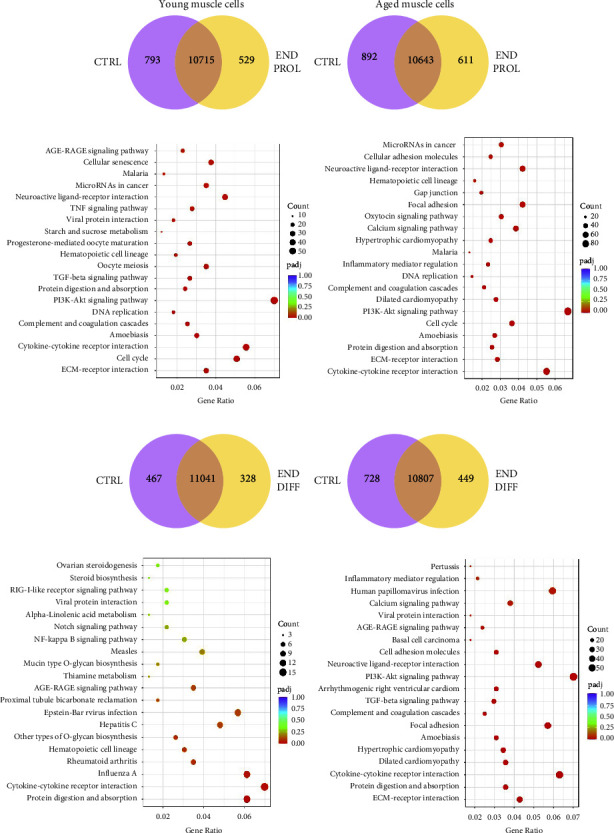
KEGG pathway analysis in human muscle cells during regeneration vs. baseline. Venn diagram of young (a) and aged (b) muscle cells showing gene transcripts exclusive to baseline (CTRL) and end of proliferation in the regeneration period (END PROL). Dot plot shows KEGG pathway enriched for different pathways in young (c) and aged (d) muscle cells at the END PROL vs. CTRL. Venn diagram of young (e) and aged (f) muscle cells showing gene transcripts exclusive to baseline (CTRL) and at the end of differentiation in the regeneration period (END DIFF). Dot plot shows KEGG pathway enriched for different pathways in young (g) and aged (h) muscle cells at the END DIFF vs. CTRL. The size of the dot is based on the gene count enriched in the pathway and the color of the dot denotes pathway enrichment significance. *n* = 4.

RNA-sequencing (RNA-seq) data were obtained from young and aged human muscle cells at preinjury (control) and during the proliferation and differentiation phases. Data obtained during proliferation or differentiation phases were compared against the baseline. Volcano plots were generated for the young and aged muscle cells. Young cells displayed 895 upregulated and 1187 downregulated genes during proliferation (Figure 4(a)), while 1447 transcripts were identified as upregulated and 2284 downregulated in aged cells (Figure 4(b)). At the end of the regeneration phase, 253 genes were significantly upregulated and 257 downregulated in young muscle cells (Figure 4(c)) and 853 upregulated and 1326 downregulated in aged cells muscle cells (Figure 4(d)) when compared with preinjury. The Venn diagram of young muscle cells during proliferation shows 10,715 gene transcripts common to baseline and 529 exclusive to proliferation (Figure 5(a)), while old muscle cells exhibit 10,643 common transcripts and 611 exclusive to proliferation (Figure 5(b)).

Kyoto Encyclopedia of Genes and Genomes (KEGG) analysis of differentially expressed genes (DEGs) identified PI3-Akt signalling, cytokine-cytokine receptor interaction, neuroactive ligand-receptor interaction, and cell cycle pathways as the most enriched in both young and older muscle cells at the end of the proliferation compared with baseline (Figures 5(c) and 5(d)).

The Venn diagram of young muscle cells during differentiation revealed 11,041 gene transcripts common between baseline and differentiation and 328 gene transcripts exclusive to differentiation, whereas the old cells exhibited 10,807 common gene transcripts and 449 exclusive to differentiation (Figures 5(e) and 5(f)).

Cytokine receptor interaction, protein digestion, and absorption together with pathways related to immune response were the most overrepresented KEGG pathways in young at the end of the differentiation period (Figure 5(g)). Aged muscle cells continued to show significant enrichment of PI3-Akt signalling pathway as well as cytokine receptor interaction and focal adhesion pathways (Figure 5(h)).

### 3.5. Aged Myotubes Depict Downregulation of Muscle Cell Differentiation and Development-Related Processes

**Figure 6:**
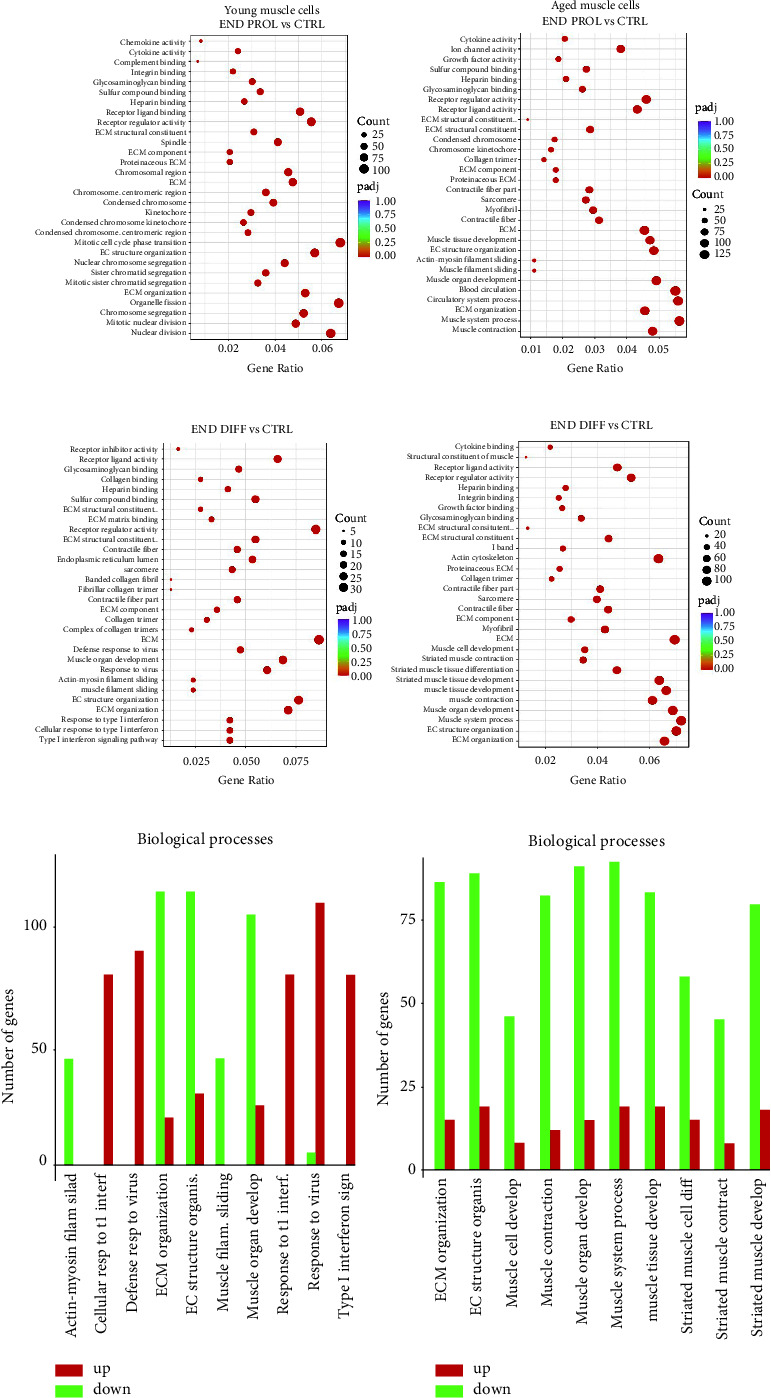
GO enrichment analysis of young and aged muscle cells show dissimilar responses. Dot plot shows overrepresented GO terms of biological processes, molecular functions, and cellular locations at the baseline (CTRL) vs. at the end of the proliferation during the regeneration period (END PROL) in young (a) and aged (b) and CTRL vs. at the end of the regeneration period (END DIFF) in young (c) and aged (d) muscle cells. The size of the dot is based on the gene count enriched in the pathway, and the color of the dot denotes pathway enrichment significance. GO enrichment bar chart of biological processes shows most enriched GO terms in young (e) and aged (f) muscle cells at the END DIFF. *n* = 4.

Gene Ontology (GO) analysis of DEGs in young myotubes identified the cell cycle as the most overrepresented molecular function during the proliferative period (Figure 6(a)). In aged ones, genes involved in muscle circulation and muscle system processes showed the highest enrichment (Figure 6(b)). At the end of the regeneration, however, extracellular matrix-related processes were the most enriched in young cells (Figure 6(c)), whereas skeletal muscle processes (i.e., muscle adaptation, contraction, hypertrophy, and relaxation) remained overrepresented in aged cells (Figure 6(d)). A closer analysis of GO enrichment demonstrates the downregulation of these biological processes in aged (Figure 6(f)) but not in young cells (Figure 6(e)), which could explain the impaired muscle cell regeneration in aged muscle shown in the morphological analysis.

## 4. Discussion

Herein, we report the successful development of a human *in vitro* model which is used to investigate the muscle regeneration response across the lifespan. This was achieved using muscle cell lines derived from both young and old donors which were then differentiated into myotubes and injured. We exposed myotubes to BaCl_2_ to induce injury and characterise the follow-on repair process with a particular focus on nuclear proliferation, muscle morphology, and the transcriptome. The transcriptome analysis was of pivotal importance since muscle regeneration relies on specific mRNA profiles for muscle reconstruction [21].

BaCl_2_ is a skeletal muscle toxin previously used to study muscle injury and regeneration *in vivo* [17, 18, 22, 23] and *in vitro* in monolayer [19] or tissue-engineered muscles [20, 24]. While the majority of the studies used animal models [8, 25, 26], only a few used human-derived muscle cells [27, 28] and none investigated the transcriptome which is fundamental to further understand muscle regeneration.

The most common method to quantify cell proliferation in response to stimuli is the BrdU assay [29, 30]. We employed EdU incorporation since in contrast to BrdU it does not require DNA denaturation which could disrupt the integrity of DNA, morphology, and antigen recognition sites. The EdU incorporation assay has been successfully used to directly measure S-phase progression in the cell cycle during the repair period following injury [8, 20]. We discovered that our muscle model retains the ability to re-enter the S-phase (proliferative) of the cell cycle as demonstrated by EdU incorporation in the repaired myotubes. Although the traditional view has been that postmitotic nuclei—like those in differentiated myocytes—do not proliferate, recent findings show otherwise. For example, a recent study where mice were labelled with green fluorescent protein and deuterium water provided evidence for proliferative activity of a myocyte [31]. Another mouse study, utilising a ^15N^thymidine stable isotope tracer, showed that cardiomyocyte nuclei proliferated in both normal and injured heart [32]. The same study confirmed that the microRNA, miR-222 was involved in the myocyte nuclei proliferation. Our findings are in line with these studies, but we appreciate that nonfused muscle cells may also have contributed to the observed proliferation and repair. This is an inherent limitation of cellular models—the maturation—and hence fusion index is never absolute. Despite this limitation, we feel that a cell model with human genetic makeup (unlike animal models) and compatible with high-throughput testing paramount, e.g., in nutrient, and drug testing offsets such as limitation.

Aged muscle cells were unable to rescue the diameter and fusion index to preinjury levels, which contrasts with young cells where full recovery was shown. These findings are in agreement with human studies examining muscle growth capacity postexercise (as a form of injury) showing an impaired hypertrophic responses in aged vs. young adults [33, 34]. Such impairment can be partly attributed to a proinflammatory systemic environment [11, 35] that may cause dysregulation of signalling pathway cascades such as FGF2, Notch, and Cdkn2a in muscle progenitor cells [25, 36, 37], resulting in a diminished differentiation capacity, evidenced herein by thinner and less fused myotubes.

We successfully mapped gene transcripts before the injury and during regeneration in young and aged human muscle cells using RNA-seq. This enabled us to identify significantly enriched KEGG signalling pathways, and GO processes evoked during muscle repair or regeneration. The KEGG analysis revealed that the cell cycle signalling pathway was significantly enriched in both young and aged muscle cells during the proliferative phase which appears to have prompted both young and aged cells to enter the proliferative S-phase after injury as discussed above.

The PI3k-Akt and downstream mTOR regulate muscle cell proliferation, survival, and differentiation [28, 38, 39]. PI3k-Akt pathway was significantly enriched in young and aged cells during the proliferation phase. When cells exited the S-phase and entered differentiation, PI3k-Akt remained enriched in aged, but not in young cells. The role of PI3k-Akt in aged cells during the differentiation phase remains somehow unclear since this pathway has many downstream effectors leading to different cell fates [39–42].

This notion is further supported by the GO analysis, which suggests that aged cells during the differentiation and proliferation exhibit significant downregulation of processes involved in muscle tissue development indicating diminished maturation capacity. Interestingly, the Venn diagram of aged muscle cells revealed that 10,807 gene transcripts overlapped between differentiation and baseline, whereas young muscle cells shared 11,041 gene transcripts between timepoints, further highlighting the difference between aged and young cells in their ability to repair to preinjury levels.

The cytokine-cytokine signalling pathway remained enriched in both young and aged cells during the proliferation and differentiation phases. Cytokines constitute a major class of regulators of skeletal myogenesis [43, 44], so its permanent overrepresentation during the recovery from injury is not surprising. Taken together, the KEGG analysis strongly suggests that factors that stimulate cell cycling played an important role during proliferation while cytokines were central across the regeneration process.

This study deserves some important considerations. For example, to reach the similar injury levels across cell lines, exposure time to BaCl_2_ in aged muscle cells had to be shorter than that in young muscle cells (2 h vs. 4 h). Although this can be considered as one of the limitations as the injury time is different across the cell lines, it also suggests that ageing diminishes the tolerance to injury and this is an interesting fact that is worth further investigations.

Another consideration relates to myotube differentiation. Although F-actin staining is commonly used to label myotubes (Bersini et al., [45]; Madden et al., [46]; Capel et al., [47]), myosin heavy chain staining could have further confirmed the maturity of the cellular muscle model.

To summarise, we successfully developed an *in vitro* model that provides a high-throughput platform enabling cellular and molecular investigations of muscle regeneration across the life course. As expected, aged myotubes showed impaired regeneration as evidenced by a reduced myofusion index and myotube width after repair. We postulate that this is due to downregulation of genes involved in muscle development and function. We anticipate the use of our model as a high-throughput platform to further examine regeneration across the lifespan, as well as to investigate potential drugs or metabolic/genetic diseases.

## Data Availability

The datasets generated and/or analysed during the current study are available in the ArrayExpress repository, E-MTAB-12248, permanent link: https://www.ebi.ac.uk/biostudies/arrayexpress/studies/E-MTAB-12248?key=4c57e37a-23c7-4c81-8d59-1f8c7e6c9373. The additional data used to support the findings of this study are available from the corresponding author (LS).
